# Diagnostic Accuracy of Xpert MTB/RIF in Sputum Smear-Negative Pulmonary Tuberculosis

**DOI:** 10.7759/cureus.5391

**Published:** 2019-08-15

**Authors:** Waqas Rasheed, Nisar Ahmed Rao, Hatem Adel, Mirza Saifullah Baig, Syed Omair Adil

**Affiliations:** 1 Pulmonology, Dow University of Health Sciences (DUHS), Karachi, PAK; 2 Radiology, Dow University of Health Sciences (DUHS), Karachi, PAK; 3 Epidemiology and Public Health, Dow University of Health Sciences (DUHS), Karachi, PAK

**Keywords:** diagnostic accuracy, smear negative, pulmonary tuberculosis, sensitivity, specificity

## Abstract

Introduction

Tuberculosis is a major health problem in Pakistan. The prevalence of pulmonary as well as extrapulmonary tuberculosis is quite high. Tuberculin skin test, radiological imaging, and sputum smear microscopy have limitations in the diagnosis of tuberculosis. Xpert MTB/RIF was recently approved for the diagnosis of pulmonary tuberculosis and has shown promising results. The aim of this study was to determine the diagnostic accuracy of Xpert MTB/RIF in sputum smear-negative pulmonary tuberculosis using acid-fast bacilli (AFB) culture as the gold standard.

Materials and methods

This cross-sectional study was conducted at Iqbal Yad Chest Clinic and Nazimabad Chest Clinic of Ojha Institute of Chest Diseases, Dow University of Health Sciences. Patients of either gender aged 18-65 years suspected to have pulmonary tuberculosis with at least two sputum samples negative for AFB underwent Xpert MTB/RIF testing. Early morning sputum samples were obtained and sent for AFB smear microscopy, Xpert testing and also for culture analysis.

Results

Mean age of the patients was 37.48 ±17.49 years. There were 84 (37.3%) females and 141 (62.7%) males. Positive findings on Xpert MTB/RIF were found in 147 (65.3%) patients whereas AFB culture showed positive findings in 174 (77.3%) patients. Sensitivity, specificity, positive predicted value, negative predicted value and overall diagnostic accuracy of Xpert MTB/RIF was found to be 84.48%, 100%, 100%, 65.38%, and 88%, respectively.

Conclusion

Xpert MTB/RIF has high sensitivity, specificity, and diagnostic accuracy in diagnosis of sputum smear-negative cases of pulmonary tuberculosis.

## Introduction

Tuberculosis (TB) is one among the major public health problems in Pakistan. The adjusted prevalence of smear-positive tuberculosis is 270 per 100,000 cases [1]. Moreover, in a study, the prevalence of sputum smear-negative culture positive tuberculosis was found to be 17.1% [2]. The conventional methods used for the diagnosis of TB like Tuberculin test/Montoux test, radiological examination and other imaging methods and sputum smear microscopy have their own limitations. Among them, acid-fast bacilli (AFB) staining of clinical material followed by smear microscopy is the most cost-effective, commonly applied microbiological test for diagnosis of TB but the smear positivity has to be supplemented with the culture positivity.

Mycobacterial culture is the gold standard, as it requires only 10-100 viable organisms to give a positive result while a minimum of 5000-10 000 AFB per ml are required for detection by smear [3]. However, culture is relatively slow and complex procedure. Solid culture usually takes four to eight weeks for results and liquid culture, though more rapid than solid culture, requires days for the detection of Mycobacterium tuberculosis. In addition, culture requires specialized laboratories and highly skilled staff.

In 2011, there has been introduction of World Health Organization (WHO) certified rapid, automated, cartridge-based nucleic acid amplification test (NAAT), the Xpert MTB/RIF assay (Cepheid, Sunnyvale, USA) (hereafter referred to as Xpert MTB/RIF), that can detect TB and rifampicin resistance simultaneously [4]. It generally produces results within two hours after starting the test, with minimal hands-on technical time. Xpert MTB/RIF detects both live and dead bacteria [5]. The sensitivity of a single Xpert MTB/RIF result in smear-positive, culture-positive cases is 98.2% whereas the sensitivity of a single Xpert MTB/RIF result in smear-negative, culture-positive cases is 72.5%; the sensitivity increases with repeated testing [6]. Xpert MTB/RIF has been found to be a cost-effective method for smear-negative TB diagnosis in comparison to traditional methods in resource-limited settings [7].

Overall Xpert MTB/RIF sensitivity was 62.6%, specificity was 99.6%, PPV was 81.3%, and NPV was 98.9% in a TB prevalence study [8]. To improve patient care and decrease TB transmission, accurate and quick detection of TB especially Smear Negative and TB drug resistance is essential. To the best of our knowledge and after going through various search engines, no published data was retrieved regarding the diagnostic accuracy of Xpert MTB/RIF in sputum smear-negative pulmonary TB (PTB) in Pakistan. Therefore, the aim of this study was to determine the diagnostic accuracy of Xpert MTB/RIF in diagnosing sputum smear-negative PTB keeping AFB culture as the gold standard.

## Materials and methods

This was a prospective cross-sectional method conducted from 1st November 2016 till 30th April 2017 at Iqbal Yad Chest Clinic (IYCC) and Nazimabad Chest Clinic (NCC) of Ojha Institute of Chest Diseases (OICD), Dow University of Health Sciences (DUHS), Karachi. Both male and female patients aged 18 to 65 years suspected to have pulmonary tuberculosis having at least 2 sputum samples negative for AFB underwent Xpert MTB/RIF testing. Patients were excluded if they were taking anti-tuberculous therapy (ATT) or were not able to expectorate. Technical laboratory-based information was collected at OICD laboratory. Early morning sputum sample i.e. sputum sample collected early morning at home next day after consultation was preferred for Xpert MTB/RIF testing. If failed to give early morning sample then spot sample i.e. sputum sample collected on the same day as the first consultation was used. Sputum volume of minimum 2 ml was considered as optimum and was processed for analysis, else rejected. The sputum sample was mixed with the Xpert sample treatment reagent at a ratio of 1:2 and transferred into the sample chamber of the cartridge. All subsequent processing steps were fully automated, in which the sample underwent concentration, amplification and detection of the rpoB DNA segment. Results were interpreted as negative if no M. tuberculosis was detected and positive if M. tuberculosis was detected with or without Rifampicin resistance as documented in the evaluation form. For culture, 0.1 ml of sediment was placed onto the Löwenstein-Jensen (LJ) medium prepared in laboratory and examined weekly up to 8 weeks for growth of Mycobacterium. Results were interpreted as positive if growth of Mycobacterium bacilli was detected and negative if no Mycobacterium bacilli growth was detected during the 8 weeks period and it was considered as the gold standard for measuring diagnostic accuracy of Xpert MTB/RIF. Data were analyzed using SPSS version 22.0. Continuous outcome variables such as age, body mass index (BMI) and duration of symptoms of the patient were mentioned as mean and standard deviation. Categorical variables like gender, findings of Xpert MTB/RIF, and AFB culture results were presented in terms of frequency and percentages. Diagnostic accuracy of Xpert MTB/RIF was assessed by calculating sensitivity, specificity, positive predictive value (PPV), and negative predictive value (NPV) using 2 x 2 table taking findings of AFB culture as the gold standard.

## Results

Total of 225 patients were included in the study. Mean age of the patients was 37.48 ±17.49 years. There were 84 (37.3%) females and 141 (62.7%) males. Mean weight, height, and BMI of the patients was 44.22 ±7.90 kg, 156.26 ±10.62 cm, and 18.09 ±2.61 kg/m2. Mean duration of symptoms was 10.40 ±6.69 days. Majority of the patients 114 (50.7%) presented with ≤10 days of duration of symptoms. General characteristics of the patients are summarized in Table 1.

**Table 1:** General characteristics of the patients

	n	%
Age, years	37.48 ±17.49^ǂ^	
≤40	138	61.3
>40	87	38.6
Gender		
Male	141	62.7
Female	84	37.3
Weight, kg	44.22±7.90^ǂ^	
Height, cm	156.26±10.62^ǂ^	
BMI, kg/m^2^	18.09 ±2.61^ǂ^	
^ǂ^mean±SD, n: number		

Xpert MTB/RIF was found positive in 147 (65.3%) patients whereas positive findings on AFB culture were seen in 174 (77.3%) patients (Table 2).

**Table 2:** Xpert MTB/RIF and culture results

Xpert MTB/RIF and culture results (n=225)			
Xpert MTB/RIF findings	AFB Culture findings		Total
	Positive	Negative	
Positive	147	0	147
Negative	27	51	78
Total	174	51	225

Sensitivity, specificity, PPV, NPV, and overall diagnostics accuracy of Xpert MTB/RIF were found to be 84.48%, 100%, 100%, 65.38%, and 88%, respectively.

## Discussion

In this study, the sensitivity, specificity, positive predictive value, negative value, and diagnostic accuracy of Xpert MTB/RIF test were performed in a highly tuberculous population.

Our study results showed that the sensitivity of Xpert MTB/RIF for diagnosis of tuberculosis is high for diagnosis of pulmonary tuberculosis. In comparison to other studies, the sensitivity is slightly lower as reported by other authors. A study from China reported a slightly higher sensitivity as compared to our population [9]. However, another study from Ethiopia has reported a lower sensitivity of this test for diagnosis of TB [10]. The sensitivity is also higher as compared to other nucleic acid tests available for detection of Mycobacterium tuberculosis [11]. This difference in sensitivities can be due to a reason of study populations and genetic differences involved. Another difference could be due to the relative different prevalence of tuberculosis among study populations.

The results of our study showed that Xpert MTB/RIF is a highly specific test for the diagnosis of tuberculosis of pulmonary origin. This high specificity has also been reported by other studies but was slightly lower as compared to our study [10-11]. Another study showed a slightly lower specificity among cases suspected to have sputum smear-negative pulmonary tuberculosis [12]. Another large multicentric study reported a high specificity of Xpert MTB/RIF for tuberculosis but slightly lower as compared to our study [13]. This difference can also be attributed to a difference in the population studied. Moreover, the quantity of an adequate sputum sample could also affect the specificity of this test. Similar specificity has been reported in population with a high prevalence of HIV [11]. Also the specificity of Xpert MTB/RIF is comparable to other commercial nuclear acid assays available [11].

Our study results showed that more than 80% of smear-negative and culture-positive cases of Mycobacterium tuberculosis were detected by Xpert MTB/RIF test. Another study showed that the detection rate of smear-negative culture positive Mycobacterium cases have been lower as compared to our study [6]. The study also showed that for improvement in sensitivity of tuberculosis detection in smear-negative cases, a second test by Xpert MTB/RIF can improve sensitivity [6].

According to the results of our study, Xpert MTB/RIF has a relatively high positive predictive value and low negative predictive value for the diagnosis of pulmonary tuberculosis. This is in contrast to an international study that has reported a high positive and negative predictive value for tuberculosis diagnosis [14].

The results of this study are also comparable to sputum samples taken by other means using Xpert MTB/RIF for tuberculosis detection. A study utilizing fiberoptic bronchoscopy sampling for early diagnosis of sputum scarce smear-negative pulmonary tuberculosis showed a comparable sensitivity and specificity to our study [15].

The results of our study should be considered in light to certain limitations. We did not evaluate drug-resistant mycobacteria in our study. Xpert MTB/RIF can also evaluate for rifampicin resistance in Mycobacterium and help as a viable tool for ruling out multi-drug resistant organisms. Another limitation of our study was that pediatric population was not studied. Studies have been carried out using Xpert MTB/RIF for diagnosis of tuberculosis in children and have shown promising results [16-17]. Not evaluating extra-pulmonary tuberculosis could also account for another limitation in our study as Xpert MTB/RIF assay can be used for its evaluation [18]. However, the sampling technique for obtaining samples of different organ system is cumbersome and different invasive or semi-invasive methods are needed for their sampling.

Despite these limitations, we believe that this study is the first study from our population for the diagnosis of smear-negative pulmonary tuberculosis. The results of this study have given insights to the diagnostic accuracy of Xpert MTB/RIF in a study setting where cost limitations and high prevalence of tuberculosis exist. In addition, prospective data were collected from all the participants involved in the current study. Short study duration can be considered as a weakness of this study.

It is recommended that further studies using Xpert MTB/RIF should be done focusing on rifampicin resistance of Mycobacteria. Moreover, a study on children should be carried out in our population to further evaluate this assay.

## Conclusions

The results of this study showed that Xpert MTB/RIF has high sensitivity, specificity and diagnostic accuracy in the diagnosis of pulmonary tuberculosis in sputum smear-negative patients.
